# The role of vitamin D against biochemical indices and oxidative stress in the heart of rats exposed to diazinon

**DOI:** 10.1515/med-2026-1410

**Published:** 2026-09-24

**Authors:** Fateme Khodaverdi, Rasool Haddadi, Mehdi Ramezani, Fereshteh Mehri, Akram Ranjbar

**Affiliations:** Department of Toxicology and Pharmacology, School of Pharmacy, Hamadan University of Medical Sciences, Hamadan, Iran; Behavioral Disorders and Substance Abuse Research Center, Institute of Neuroscience and Mental Health, Avicenna Health Research Institute, Nutrition Health Research Center, Hamadan University of Medical Sciences, Hamadan, Iran; Department of Pharmacology Toxicology, School of Pharmacy Medicinal Plants and Natural Products Research Center, Hamadan University of Medical Sciences, Hamadan, Iran; Department of Anatomical Sciences, School of Medicine, Hamadan University of Medical Sciences, Hamadan, Iran

**Keywords:** diazinon, vitamin D, heart, oxidative stress

## Abstract

**Objectives:**

Diazinon is used as an organophosphate for pest management in agriculture and veterinary practice. However, diazinon poisoning can lead to increased production of free radicals and destruction of the body’s antioxidant system. A study explored the impact of vitamin D on diazinon-induced cardiotoxicity in rats.

**Methods:**

24 male Wistar rats were randomly assigned to four groups: a control group treated with corn oil, a vitamin D group receiving 0.2 mg/kg/ip/day, and a DZN group receiving 70 mg/kg/po/day. Additionally, treatment groups were formed that received both DZN (70 mg/kg/po/day) and vitamin D (0.2 mg/kg/ip/day). On the final day of the experiment, the rats were euthanized, and their blood was analyzed for serum levels of troponin (TPI) and creatine kinase (CK). Furthermore, the heart tissue’s concentrations of superoxide dismutase (SOD), catalase (CAT), total antioxidant capacity (TAC), total thiol groups (TTG), and malondialdehyde (MDA) were assessed. Hematoxylin and eosin (H&E)-stained slides were also used to analyze histopathological alterations in the cardiac tissue.

**Results:**

DZN significantly increased the levels of TPI, CK, and MDA, which may indicate cardiac damage. In addition, DZN decreased the levels of CAT, TAC, SOD, and TTG. On the contrary, vitamin D significantly reduced oxidative damage and increased antioxidant activities in animals with DZN. Also, these findings had a good correlation with histopathological changes.

**Conclusions:**

Vitamin D, as an antioxidant, enhances antioxidant activities in heart tissue; it effectively prevents oxidative damage caused by DZN.

## Introduction

Organophosphate (OP) toxins are a persistent and dangerous component of pesticides in industrial and agricultural fields [1]. Their widespread use leads to environmental pollution and increases the risk of toxicity in both acute and chronic forms by inhibiting acetylcholinesterase and enhancing the production of free radicals, which, as a result of free radical release, alter the function of antioxidant enzymes and glutathione levels [2]. It reduces and increases lipid peroxidation in the organism [3]. Diazinons (DZN), a subgroup of organophosphate pesticides, are characterized by their phosphorus content derived from phosphoric acid and are often used as insecticides [4]. The predominant mode of DZN-induced toxicity in animal models is attributed to the inhibition of acetylcholinesterase mediated by the diazoxone metabolite, which has been documented to lead to oxidative stress in vital organs of rats, including the brain, heart, and spleen [5]. DZN in rodents has decreased the level of red blood cells, hemoglobin concentration, and closed cell volume in serum and biochemical parameters [6]. Toxicities related with OP may manifest as sinus tachycardia, sinus bradycardia, hypertension, hypotension, impaired heart rate and force of contraction. ECG changes associated with OP include prolonged QTc interval, ST segment elevation, low amplitude T waves, and prolonged PR interval [7]. DZN also reduced myocardial contractility and precipitated left ventricular hypertrophy with resultant heart failure. These results thus corroborate the report of Boroushaki et al. and Cakici and Akat [8], [9].

Vitamin D is a fat-soluble prohormone [10]. When skin is exposed to UV light, it converts 7-dehydrocholesterol into vitamin D [11]. This vitamin can also be absorbed by the digestive system [12]. The active form of vitamin D, known as calcitriol (1,25(OH)_2_D), is crucial for the production and secretion of insulin, as well as for maintaining calcium balance in beta islet cells [13]. The liver enzyme 25-hydroxylase converts 7-dehydrocholesterol into calcidiol (25(OH)D) [14]. Subsequently, the kidney’s 1-alpha hydroxylase enzyme converts calcidiol into 1,25-dihydroxyvitamin D (1,25(OH)_2_D) [15]. Vitamin D insufficiency is associated with reduced insulin sensitivity, decreased insulin circulation, and poor glucose tolerance [16]. Exercising anti-inflammatory and anti-fibrotic effects [17], the renin-angiotensin system [18], cancer [18], infectious diseases [19], and diabetes and metabolic syndrome [20] are interrelated. Vitamin D inhibits the proliferation of T cells and the transcription of inflammatory cytokines by VDR (vitamin D receptor) [21], 22]. If the vitamin D receptor is damaged, it stimulates the renin-angiotensin system and high blood pressure, increasing the concentration of the ANP (Atrial Natriuretic Peptide) hormone and heart muscle hypertrophy [23]. Oxidative imbalance has been associated with the progression of atherosclerosis and cardiovascular outcomes, and vitamin D may be effective in reducing cardiac complications due to its ability to reduce oxidative stress through upregulation of cellular glutathione and SOD [24]. Prospective studies have shown a correlation between hypovitaminosis D, increased levels of oxidative stress markers, and significant thickening of the common carotid intima. Pathophysiological mechanisms proposed for this association with the relative risk of AH include the deleterious effects of hypovitaminosis D on endothelial function [25]. According to the published report and the importance of vitamin D in protecting heart tissue, this study aims to investigate the protective effects of vitamin D3 as an antioxidant in treating subacute toxicity with diazinon in rat heart tissue.

## Materials and methods

### Chemicals

All the materials for this study were purchased from Merck (Germany), except those mentioned. Ketamine and xylazine were obtained from Alfasan (Netherlands).

### Animal

For this investigation, twenty-four male Wistar rats weighing 180 and 230 g were obtained from the animal center laboratory at Hamedan University of Medical Sciences (HUMS). The rats were kept under standard laboratory conditions, which included a 12-h light and 12-h dark photoperiod, a controlled temperature of 25 ± 2 degrees Celsius, and unrestricted access to food and water. Before the experimental procedures, the subjects underwent a one-week acclimatization period in the laboratory environment. The research protocol received approval from the ethics committee of HUMS, as indicated by the ethical approval code IR. UMSHA. REC.1401.767.

### Experimental groups

In this study, 24 rats were randomly divided into four six groups and observed for 28 days. The groups included: a control group (administered corn oil orally), a diazinon group (70 mg/kg/day, orally), a vitamin D group (0.2 mg/kg/day, intraperitoneally), and a diazinon + vitamin D group (administered 70 mg/kg/day, orally, and 0.2 mg/kg/day, intraperitoneally) [26], [27], [28]. The rats in each group were placed under anesthesia throughout the study, receiving an intraperitoneal injection of 10.80 mg/kg of ketamine/xylazine. After a laparotomy, blood samples were collected from the inferior vena cava (IVC) and then centrifuged at 3,000 rpm for 10 min. The obtained serum was stored at −20 °C for biomarker evaluation. The hearts were preserved, with a portion of the heart tissue rinsed with cold phosphate-buffered saline (PBS), immediately frozen in liquid nitrogen, and stored at −80 °C for biochemical analysis. Another portion of the heart tissue was also preserved in 10 % formalin for future histological experiments [27].

### Serum biochemical analysis

Serum activity of creatine phosphokinase (CPK) and serum indicators such as troponin (TPI) were measured using diagnostic kits purchased from Biorex Fars (Iran).

### Oxidant/antioxidant markers

Assessment of liver oxidative damage LPO, TAC, CAT, SOD, and TTG were measured with the available kit. As the final product of lipid peroxidation and an indicator of oxidative stress, MDA was measured through di thiobarbituric acid (TBA) and absorption measurement at a wavelength of 532 nm, and the results were expressed as protein nmol/mg. The TAC level was measured by creating a blue color complex and reducing Fe^3+^ to Fe^2+^ using TPTZ (Tripyridyl-s-triazine). The complex’s absorption was analyzed at 593 nm.

CAT activity was measured by measuring the rate at which H_2_O_2_ disappeared at 240 nm. The CAT levels in homogenous tissue were expressed as U/mg pr. The xanthine oxidase reaction was used to measure the number of superoxide radicals generated by 2-(4-iodophenyl)-3-(4-nitrophenol)-5-phenyl tetrasodium chloride, and this allowed for the assessment of SOD activity. At a wavelength of 550 nm, the absorbance of this combination was measured, and the results were represented as U/gr protein. DTNB was used as a reagent, and the tissue level of TTG was determined. A yellow compound was produced when the DTNB and thiol functional groups interacted. TTG levels were expressed as µmol/mg protein, and absorbance was measured at a wavelength of 412 nm [26], [27].

### Histological studies

The cardiac specimens were fixed in a 10 % buffered formalin solution, embedded in paraffin, and carefully sectioned into 5 μm thick slices. Afterward, they were stained using Masson’s trichrome and hematoxylin and eosin (H&E) and analyzed with an Olympus CX31 light microscope at a 40x magnification [27].

### Statistical analysis

The data is presented as the mean plus or minus the standard deviation (Mean ± SD). We used SPSS Version 16.0 to determine the mean difference between groups, employing a one-way analysis of variance (ANOVA) test followed by Tukey’s post hoc test. If the p-value is less than 0.05, the statistical result can be considered significant [29].

## Ethical approval

The research protocol received approval from the ethics committee of HUMS, as indicated by the ethical approval code IR. UMSHA.REC.1401.767.

## Results

### The effect of DZN and vitamin D on serum biochemistry

**Figure 1:**
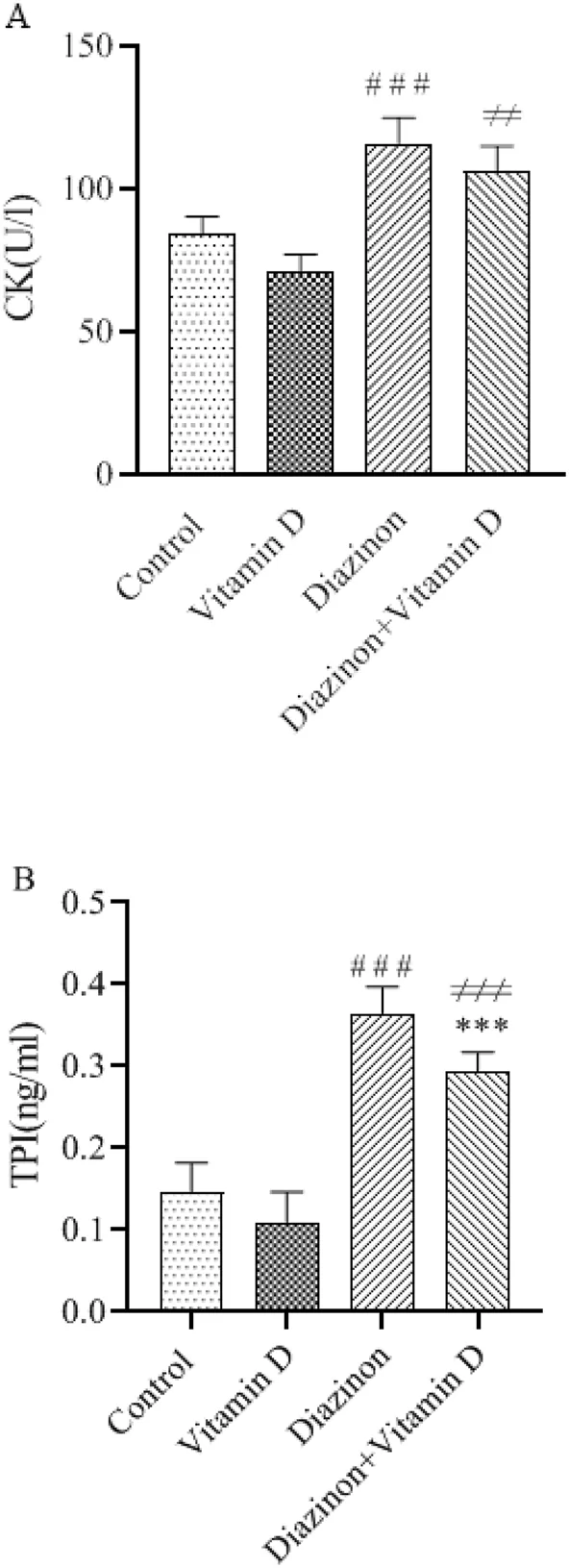
The effect of vitamin D on parameters A: TPI (troponin) and B: CK (creatine kinase) was reported. Data were expressed as mean ± SD ^###^p<0.001 compared to the control group, ^≠≠≠^p<0.001 and ^≠≠^p<0.01 compared to the vitamin D group. ^***^p<0.001 compared to diazinon group (n=6).

The serum activity of the CK enzyme was significantly increased (p<0.001) in the group treated with Diazinon compared to the control group. In the Diazinon-poisoned group, administration of vitamin D resulted in a significant increase in serum CK levels compared to the group that received only vitamin D (p<0.01) (Figure 1A). Additionally, serum TPI activity in the Diazinon group was significantly higher than that in the control group (p<0.001) (Figure 1B). Notably, there was a significant relationship (p<0.001) between serum TPI activity in the group treated with both vitamin D and Diazinon, compared to the group treated with only vitamin D (which showed an increase) and the group treated with only Diazinon (which showed a decrease).

### Measurements of oxidative stress biomarker

#### Effect of vitamin D and diazinon on LPO levels

**Figure 2:**
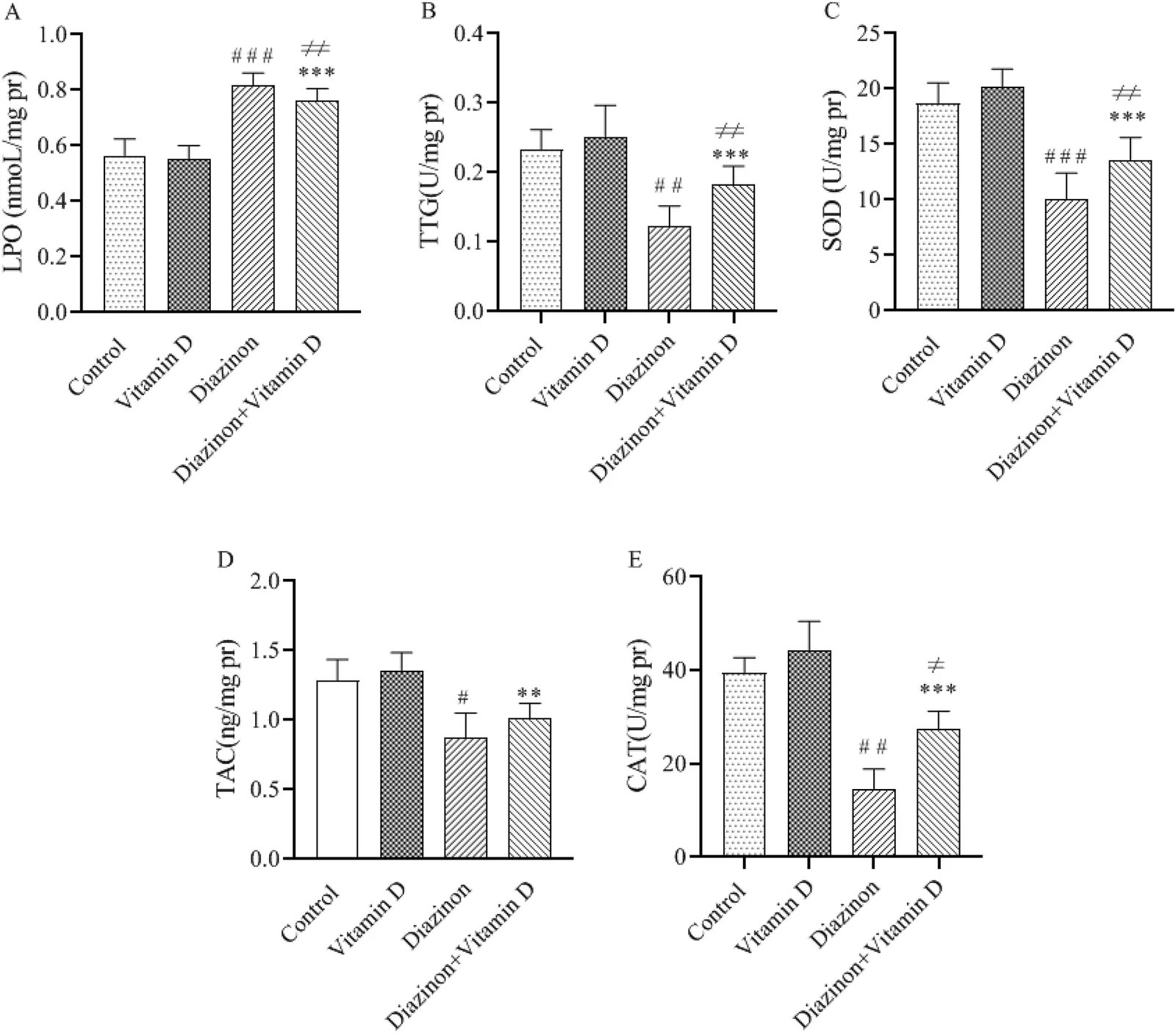
Effects of vitamin D on the heart LPO (A), TTG (B), SOD (C), TAC (D), and CAT (E) levels in samples of poisoned rats by diazinon. The data were reported using mean ± SD (n=6). ^#^p<0.05, ^##^p<0.01 avd ^###^p<0.001 compared to control group, **p<0.01 and ***p<0.001 compared to diazinon group. ^*≠*^p<0.05, ^*≠≠*^p<0.01 avd ^*≠≠≠*^p<0.001 compared to vitamin D group.

The level of lipid peroxidation (LPO) in the heart samples significantly increased in the diazinon group compared to the control group (p<0.001). In the diazinon-poisoned group that received vitamin D treatment, there was a notable decrease in LPO levels compared to the diazinon group (p<0.001). However, the LPO levels in the hearts of the diazinon-poisoned group with vitamin D treatment remained higher than those in the vitamin D-only group (p<0.01) (Figure 2A).

#### Effect of vitamin D and diazinon on TTG levels

The TTG level in the heart samples was significantly lower in the diazinon group compared to the control group (p<0.01). Treatment with vitamin D in the diazinon patient group significantly increased the TTG level compared to the diazinon group alone (p<0.001). However, the administration of vitamin D to the diazinon group resulted in a decrease in the TTG level in the heart when compared to the vitamin D group (p<0.01) (Figure 2B).

#### Effect of vitamin D and diazinon on SOD levels

The levels of superoxide dismutase (SOD) in the diazinon group decreased significantly when compared to the control group (p<0.001). Animals in the diazinon group who received vitamin D treatment showed a significant increase in SOD levels compared to those who only received diazinon (p<0.001). However, the group that was poisoned with diazinon and treated with vitamin D exhibited a decrease in SOD levels in the heart compared to the vitamin D-only group (p<0.01) (Figure 2C).

#### Effect of vitamin D and diazinon on TAC levels

In the diazinon group, the total antioxidant capacity (TAC) level was significantly lower compared to the control group (p<0.05). However, animals in the diazinon group treated with vitamin D exhibited a significant increase in TAC levels compared to those solely receiving diazinon (p<0.05). The administration of vitamin D and diazinon treatment resulted in higher heart TAC levels than those in the diazinon group alone (p<0.01). Nevertheless, the group that was poisoned with diazinon and treated with vitamin D did not show a significant decrease in heart TAC levels when compared to the vitamin D-only group (p>0.05) (Figure 2D).

#### Effect of vitamin D and diazinon on CAT levels

**Figure 3:**
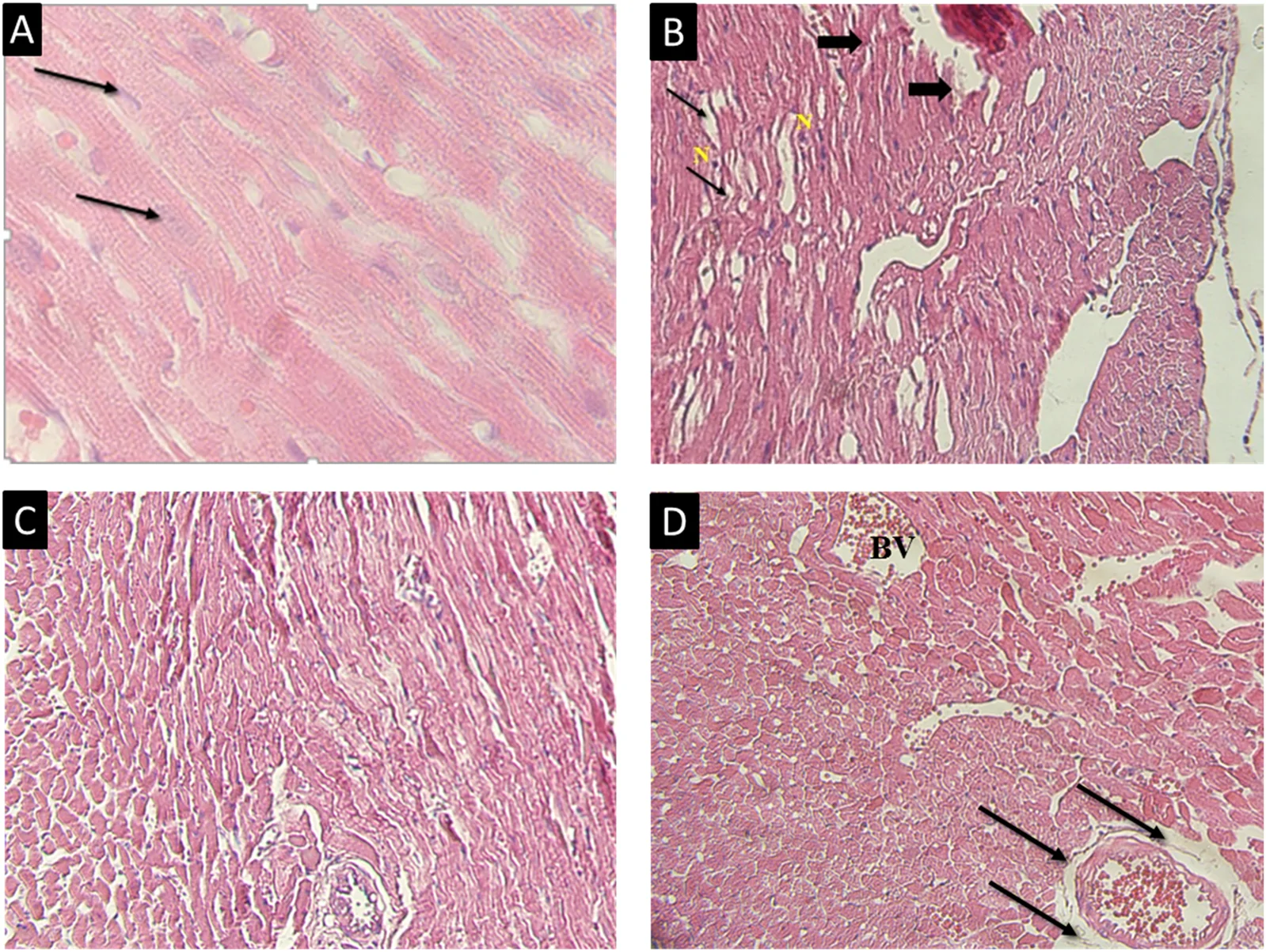
Effect of vitamin D on diazinon-induced heart histopathological changes. Control groups (A), diazinon (B), vitamin D (C), diazinon + vitamin D (D). Control group showed normal histological appearance of cardiac myocyte and normal nucleus (arrows). Diazinon group was showed vacuolated sarcoplasm (thin arrows), degeneration of muscle fibers (thick arrows), pyknotic nuclei (n). There is increased interstitial space in places (lighter “gaps” between fibres) suggesting possible oedema or mild separation of fibres. The group Vit. D showed cardiac muscle fiber of normal shape size and configuration. Diazinon+ Vit. D of male rat showed: sub capsular congestion of blood vessels (arrows), dilatation and congestion of blood vessels (BV), loss of myofibril integration. A: 400× and B: 200× and C, and D with magnification of 100×.

When compared to the control group, the level of catalase (CAT) was significantly lower in the diazinon group (p<0.001). The patient group that received vitamin D in addition to diazinon showed a significant increase in CAT levels compared to the group that only received diazinon (p>0.001). However, the diazinon-poisoned group treated with vitamin D still exhibited lower cardiac CAT levels than the vitamin D-only group (p<0.05) (Figure 3).

### Histological results

**Figure 4:**
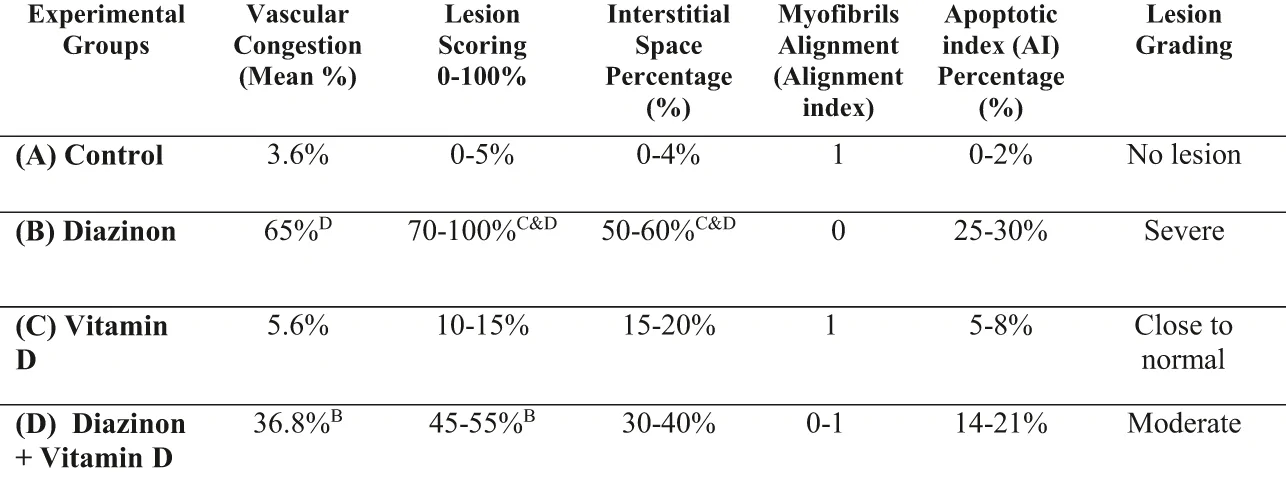
Histological a blinded semi-quantitative scoring of heart. Area of vascular congestion was estimated by (µm). Each value represents mean ± SDM (n=6). Statistical comparison among groups: mean values with different capital letters have significant differences at (p‹0.05). Alignment index: numerical index of how aligned the fibers are (1=perfectly aligned, 0=random). Apoptotic index (AI): number of apoptotic cells per total cells counted (%). (A) Control group, (B) diazinon, (C) vitamin D, (D) diazinon and vitamin D.

The histopathological study of the heart muscle in the control group shows the normal histological appearance of the cardiac myocyte and the normal nucleus (arrow) (Figure 4A). In the diazinon-treated group, disarrangement of muscle fibers shows vacuolated sarcoplasm (thin arrows), degeneration of muscle fibers (thick arrows), and pyknotic nuclei (n) (Figure 4B). The vitamin D group shows cardiac muscle fibers of normal size and configuration (Figure 4C). Under treatment with diazinon and vitamin D, subcapsular congestion of blood vessels (arrow), dilation and congestion of blood vessels (BV), and loss of myofibril integrity were observed (Figure 4D).

## Discussion

The heart tissue requires a continuous supply of oxygen and nutrients to reduce oxidative stress due to its functional importance and limited antioxidant enzyme activity [30]. In the present study, we found that vitamin D may be crucial in protecting the heart from oxidative stress caused by DZN [24]. LPO is one of the most important and standard markers of lipid peroxidation; the increase in its level indicates a disturbance in the defense mechanisms of non-enzymatic and enzymatic antioxidants [31]. In the current study, diazinon at a dose of 70 mg/kg significantly increased the concentration of LPO compared to the control group, which led to peroxidation of membrane lipids and oxidative stress. LPO is an important aldehyde resulting from the peroxidation of membrane lipids [32]. Studies indicate that the oral administration of dimethoate, methyl parathion, and malathion in rats alters the activity of antioxidant enzymes and increases lipid peroxidation in erythrocytes [33]. Ogutcu et al. demonstrated that DZN elevates the level of MDA in the heart tissue of rats, indicating the generation of free radicals in this organ [34]. According to research conducted by Tahamasbi et al. it was found that vitamin E decreases the level of malondialdehyde and increases glutathione levels compared to the DZN group, which is attributed to the inhibitory effects of vitamin E on oxidative stress and tissue fat peroxidation [35]. The results of our study support the importance of vitamin D in reducing the production of TBARS. Our findings demonstrate the antioxidant capacity of vitamin D, particularly in inhibiting iron-dependent lipid peroxidation in liposomes.

The TTG system can participate directly or as a substrate of glutathione peroxidase and glutathione S-transferase enzymes in detoxifying hydrogen peroxide, lipid hydroperoxides, and electrophilic compounds [36]. Depletion of glutathione may lead to increased lipid peroxidation, DNA damage, developmental arrest, and reduced resistance to oxidative damage [32]. The involvement of vitamin D can explain the decrease in TTG content in rats when exposed to DZN as an antioxidant that fights free radicals. The present study shows that the administration of diazinon decreases the concentration of TTG compared to the control group of heart tissue. This reduction of TTG is probably due to the increase in the activity of enzymes such as glutathione peroxidase and GST, which require TTG [37]. In the study conducted by Isik et al., the introduction of methyl parathion and DZN to fish led to a decrease in TTG levels in liver tissue [38]. Birdane et al. showed that DZN increases the activity of TTG in the spleen tissue, which indicates an increase in the organism’s defense against toxins and its rapid elimination [39].

Enhancing the endogenous antioxidant activity could help reduce oxidative stress in heart tissue [30]. This study found that the administration of diazinon increased the activity of the SOD and CAT enzymes in the hearts of rats compared to the control group. Additionally, the administration of vitamin D partially reduced the activity of these enzymes when compared to the diazinon group. By increasing free radicals, diazinon activates antioxidant defense systems, including SOD and CAT enzymes [40]. The increase in SOD activity in this study decreases the superoxide radical and increases H2O2 in heart tissue, and the increase in catalase enzyme activity neutralizes H2O2 [41]. The decrease in the activity of SOD and CAT enzymes after vitamin D administration is probably related to their ability to directly eliminate ROS [42]. Almost throughout the study, there was a significant difference in stress levels and antioxidant parameters, except for TAC in the diazinon-poisoned group treated with vitamin D, compared to the vitamin D-only group. It was shown by Akturk et al. that the increase in MDA level and the activity of catalase and superoxide dismutase in the heart decreased with the simultaneous administration of DZN and vitamins C and E [43]. Another study by Hassani and colleagues showed that diazinon decreased the levels of antioxidant enzymes, including SOD-CAT, which was consistent with this research [40]. There is a growing body of evidence which reveals the impact of VD in ameliorating the induced OS and inflammatory damages by activating the Nrf2 signaling pathway and regulating the NF-κB pathway. It can be stated that Nrf2 is a master regulator of the antioxidant defense system and its increased expression occurs as a result of oxidative stress. This is a common cellular response to combat OS and leads to the activation of phase II enzymes including SOD and CAT [44].

Creatine kinase (CK) is an enzyme found mainly in the heart and skeletal muscles, and in small amounts in the human brain [45]. When damaged, skeletal muscle, cardiac muscle, or brain tissue releases creatine kinase into the bloodstream. Even though diazinon raises CK activity in plasma, elevated levels of this enzyme in plasma suggest temporary injury to other tissues, such as muscle fibers [46]. According to Banaee et al.’s study, CK levels significantly increased with prolonged exposure to diazinon doses [47]. Studies have shown that vitamin D is associated with reduced TPI and may help mitigate diazinon-induced cardiac inflammation [48]. In Kouchek’s study, improved vitamin D status was associated with reduced inflammation and improved cardiac function, which may contribute to lower CK-MB levels [49]. Histopathological observations showed that mice treated with DZN showed disorganization of muscle fibers, vacuolated sarcoplasm, degeneration of muscle fibers, and pyknotic nuclei. Diazinon is associated with producing free radicals, which are also involved in many pathophysiological processes [46]. On the contrary, administration of vitamin D significantly improved the effects of oxidative stress in heart tissue, and our results were consistent with the histological observations made by Salemet al [50]. This aligns with the findings of Gül and Aygü, who found that vitamin D supplementation decreased cardiac marker enzymes in rats with doxorubicin-induced cardiotoxicity [51]. Animal studies have shown that vitamin D can reduce myocardial fibrosis through signaling pathways including changes in mRNA expression of cardiac atrial natriuretic peptide (ANP), matrix metalloproteinase-2 (MMP-2), matrix metalloproteinase-9 (MMP-9), and tissue inhibitor of metalloproteinase-1 (TIMP-1) expression [52].

## Conclusions

Diazinon causes oxidative stress in the heart tissue by producing free radicals and changing the function of antioxidant enzymes. Because vitamin D, as an antioxidant, enhances antioxidant activities in heart tissue, it effectively prevents oxidative damage caused by DZN.
